# A guide to selecting high-performing antibodies for Alpha-1-antitrypsin (UniProt ID: P01009) for use in western blot, immunoprecipitation and flow cytometry

**DOI:** 10.12688/f1000research.175969.1

**Published:** 2026-01-19

**Authors:** Jemma Cooper, Carolyn Jones, Katie Dixon, Bibek Gooptu, Harvinder Virk, Michael Biddle

**Affiliations:** 1 University of Leicester College of Life Sciences, Leicester, England, UK; 2 Institute for Precision Health, University of Leicester, Leicester, England, UK

**Keywords:** P01009, alpha-1-antitrypsin, SERPINA1, A1AT, serine protease inhibitor, antibody validation, western blot, immunoprecipitation, flow cytometry

## Abstract

Alpha-1-antitrypsin (A1AT), encoded by the *SERPINA1* gene, is a circulating serine protease inhibitor that protects the lung from neutrophil elastase–mediated tissue damage. Mutations in *SERPINA1* cause alpha-1-antitrypsin deficiency, one of the most common hereditary causes of respiratory disease, leading to early-onset emphysema and chronic obstructive pulmonary disease, as well as hepatic complications. Given the clinical significance of A1AT and its widespread use as a biomarker, there is a critical need for high-quality antibodies to ensure reliable detection and mechanistic insights. Here we systematically characterised eighteen commercial antibodies for western blot, immunoprecipitation, and flow cytometry using a standardized knockout validation approach in human Hep G2 cells, comparing readouts in a *SERPINA1* knockout line with its isogenic parental control. This work is part of a broader collaborative initiative aimed at addressing antibody reproducibility by characterising commercial antibodies for human proteins and openly sharing the results with the community. While antibody use and protocols vary between laboratories, we encourage readers to use this report as a guide to select the most appropriate antibodies for their specific experimental needs.

## Introduction

Alpha-1-antitrypsin (A1AT) deficiency is a hereditary disorder caused by mutations in the *SERPINA1* gene, leading to reduced circulating levels of functional A1AT, a serine protease inhibitor primarily synthesized in the liver. ^1^ Under normal conditions, A1AT diffuses into the lungs where it neutralises neutrophil elastase, thereby preserving the protease–antiprotease balance essential for alveolar integrity. ^1, 2^ In individuals with A1AT deficiency, insufficient inhibition of neutrophil elastase results in progressive alveolar wall destruction and panacinar emphysema, particularly in the lower lobes of the lungs. ^2, 3^ Clinically, this manifests as early-onset emphysema, chronic obstructive pulmonary disease (COPD), and chronic bronchitis, with disease severity strongly influenced by environmental factors such as cigarette smoking. ^4, 5^

In addition to pulmonary complications, A1AT deficiency has important hepatic consequences. Mutant A1AT proteins, such as the Z allele (Glu342Lys), misfold within the endoplasmic reticulum of hepatocytes and form toxic aggregates that are poorly secreted into circulation. ^1, 6^ This intracellular accumulation predisposes to a spectrum of liver disease and increases the risk of hepatocellular carcinoma in adulthood. ^6^

For the robust assessment of A1AT biology, the use of well-characterised experimental tools is essential. Given the widespread use of immunoassays and immunohistochemistry to detect A1AT expression, ^7, 8^ antibody validation is critical to ensure specificity and reproducibility. Poorly validated antibodies can lead to misleading findings regarding A1AT levels, distribution, and misfolding, thereby obscuring the disease mechanisms and hindering the development of effective diagnostics and therapies. ^9– 11^

This research is part of a broader collaborative initiative in which academics, funders and commercial antibody manufacturers are working together to address antibody reproducibility issues by characterising commercial antibodies for human proteins using standardized protocols ^12^ and openly sharing the data. ^13^ Here we evaluated the performance of eighteen commercial antibodies for A1AT for use in western blot, immunoprecipitation and flow cytometry, enabling biochemical and cellular assessment of A1AT properties and function. The platform for antibody characterisation used to carry out this study was endorsed by a committee of industry academic representatives. It consists of identifying human cell lines with adequate target protein expression and the development/contribution of equivalent knockout (KO) cell lines, followed by antibody characterisation procedures using most commercially available renewable antibodies against the corresponding protein. The standardised consensus antibody characterisation protocols are openly available on Protocols.io (DOI: dx.doi.org/10.21203/rs.3.pex-2607/v1).

The authors do not engage in result analysis or offer explicit antibody recommendations. Our primary aim is to deliver top-tier data to the scientific community, grounded in Open Science principles. This empowers experts to interpret the characterisation data independently, enabling them to make informed choices regarding the most suitable antibodies for their specific experimental needs. Guidelines on how to interpret the antibody characterisation data found in this study are featured on the YCharOS gateway. ^14^

## Results and discussion

Our standard protocol involves comparing readouts from WT (wild type) and KO cells. ^15, 16^ The first step is to identify a cell line(s) that expresses sufficient levels of a given protein to generate a measurable signal using antibodies. To this end, we examined the DepMap transcriptomics database to identify all cell lines that express the target at levels greater than 2.5 log _2_ (transcript per million “TPM” + 1), which we have found to be a suitable cut-off (Cancer Dependency Map Portal, RRID:SCR_017655). The cell line Hep G2 expresses the *SERPINA1* transcript at 12.5 log _2_ (TPM+1) and thus was identified as a suitable cell line and was modified by CRISPR/Cas9 to KO the corresponding *SERPINA1* gene ( Table 1).

**Table 1.** Summary of the cell lines used.

Institution	Catalogue number	RRID (Cellosaurus)	Cell line	Genotype
Sigma Aldrich	85011430-1VL	CVCL_0027	HepG2	WT
University of Leicester	-	CVCL_F0RL	HepG2	*SERPINA1* KO

**Figure 1.**
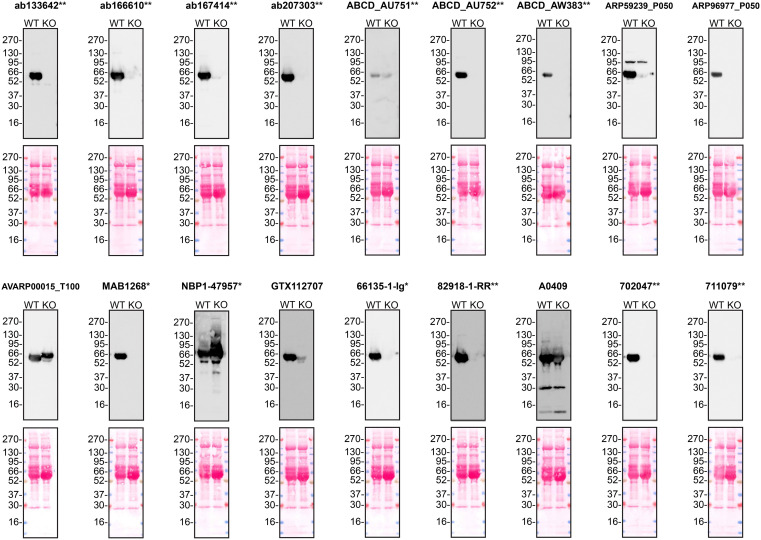
Alpha-1-antitrypsin antibody screening by western blot using concentrated conditioned culture media. Culture media from Hep G2 WT and *SERPINA1* KO cells were collected, and 30μg of protein was processed for western blot with the indicated Alpha-1-antitrypsin antibodies. The Ponceau stained transfers of each blot are presented to show equal loading of WT and KO samples. Antibody dilutions were chosen according to the recommendations of the antibody supplier. Antibody dilutions used: ab133642** at 1/1000, ab166610** at 1/1000, ab167414** at 1/1000, ab207303** at 1 in 5000, ABCD_AU751** at 1/5, ABCD_AU752** at 1/85, ABCD_AW383** at 1/35, ARP59239_P050 at 1/500, ARP96977_P050 at 1/500, AVARP00015_T100 at 1/800, MAB1268* at 1/500, NBP1-47957* at 1/1000, GTX112707 at 1/3000, 66135-1-Ig* at 1/5000, 82918-1-RR** at 1/2000, A0409 at 1/15000, 702047** at 1/500 and 711079** at 1/500. Predicted band size: 46.7 kDa. *Monoclonal antibody; **Recombinant antibody.

**Figure 2.**
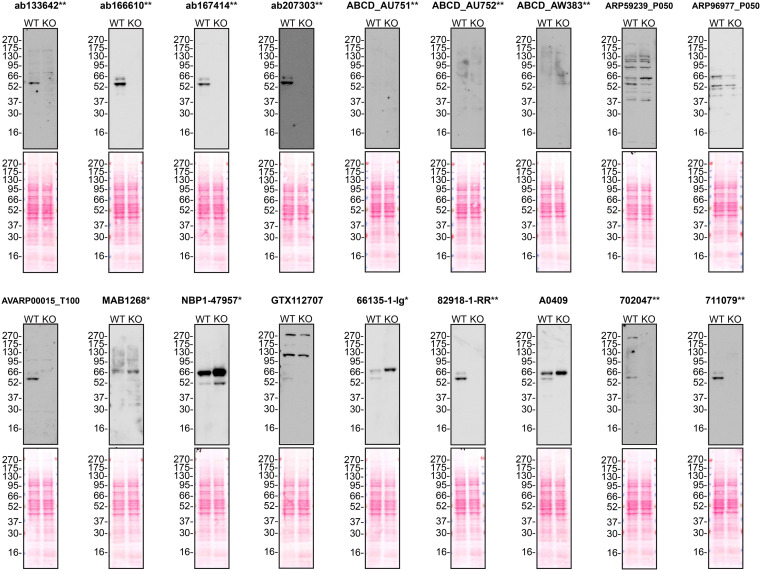
Alpha-1-antitrypsin antibody screening by western blot using protein lysates. Protein lysates from Hep G2 WT and *SERPINA1* KO cells were collected, and 30μg of protein was used for western blot with the indicated Alpha-1-antitrypsin antibodies. The Ponceau stained transfers of each blot are presented to show equal loading of WT and KO samples. Antibody dilutions were chosen according to the recommendations of the antibody supplier. Antibody dilutions used: ab133642** at 1/1000, ab166610** at 1/1000, ab167414** at 1/1000, ab207303** at 1 in 5000, ABCD_AU751** at 1/5, ABCD_AU752** at 1/85, ABCD_AW383** at 1/35, ARP59239_P050 at 1/500, ARP96977_P050 at 1/500, AVARP00015_T100 at 1/800, MAB1268* at 1/500, NBP1-47957* at 1/1000, GTX112707 at 1/3000, 66135-1-Ig* at 1/5000, 82918-1-RR** at 1/2000, A0409 at 1/15000, 702047** at 1/500 and 711079** at 1/500. Predicted band size: 46.7 kDa. *Monoclonal antibody; **Recombinant antibody.

According to the UniProt database, A1AT is secreted into the extracellular space. As such, clarified concentrated medium from both Hep G2 WT control and *SERPINA1* KO cell lines was run on SDS-PAGE, transferred onto nitrocellulose membranes and probed in parallel with eighteen A1AT antibodies ( Figure 1). Antibodies were then assessed for their ability to detect intracellular A1AT using protein lysates from WT control and *SERPINA1* knockout cells ( Figure 2). A1AT expression was detected in the protein lysates but required a longer exposure time, indicating that A1AT is predominantly secreted by Hep G2 cells. Therefore, the performance of antibodies by immunofluorescence was not evaluated in this study.

**Figure 3.**
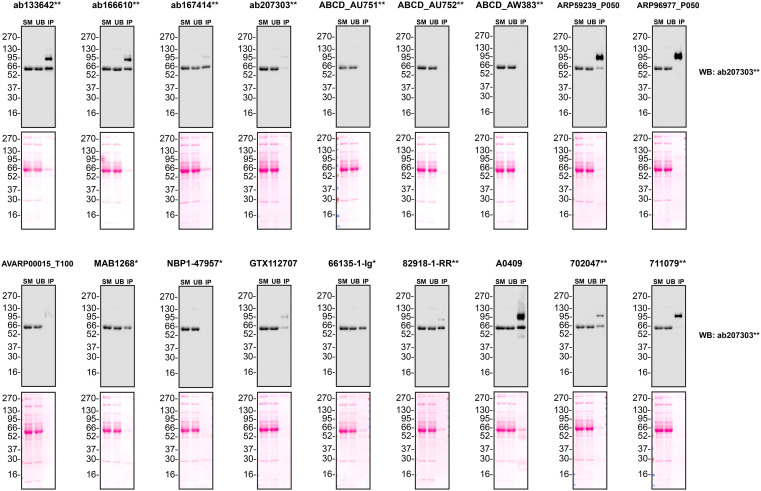
Alpha-1-antitrypsin antibody screening by immunoprecipitation of culture medium. Conditioned culture medium was collected from Hep G2 WT cells, and immunoprecipitation was performed for 18 hours using 0.5 mg of protein and 2.0 μg of the indicated Alpha-1-antitrypsin antibodies pre-coupled to Dynabeads protein A or protein G. Samples were washed and processed for western blot with the anti- Alpha-1-antitrypsin antibody ab207303** diluted at 1/5000. The Ponceau stained transfers of each blot are shown. SM=4% starting material; UB = 4% unbound fraction; IP = immunoprecipitate. *Monoclonal antibody; **Recombinant antibody.

We next assessed the ability of all eighteen antibodies to capture A1AT from Hep G2 culture medium by immunoprecipitation, followed by western blot analysis. For the immunoblot, a specific A1AT antibody identified previously ( Figure 1) was used. Equal amounts of the starting material (SM) and unbound fraction (UB), along with the complete immunoprecipitated (IP) eluate, were separated by SDS-PAGE ( Figure 3).

**Figure 4.**
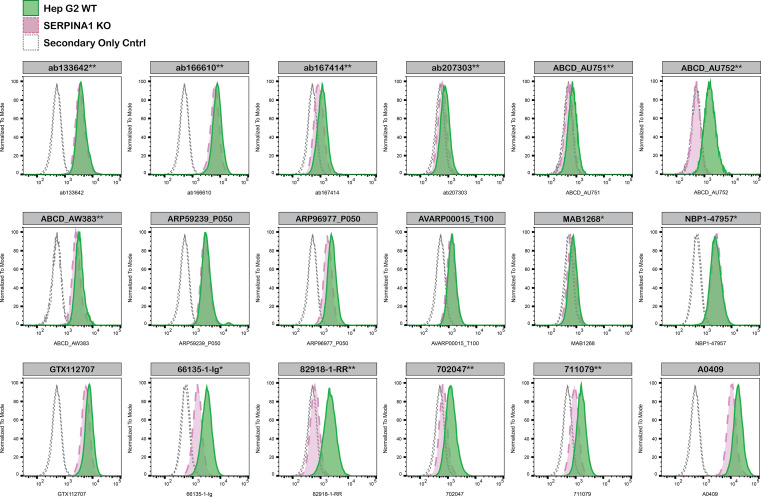
Alpha-1-antitrypsin antibody screening by flow cytometry. Hep G2 WT and *SERPINA1* KO cells were labelled with a green or violet, fluorescent dye, respectively. WT and KO cells were mixed in a 1:1 ratio, fixed in 4% PFA and permeabilized in 0.1% saponin. 400,000 cells were stained with the indicated alpha-1-antitrypsin antibodies and corresponding Multi-rAb CoraLite ^®^ Plus 647 secondary antibodies. Antibody staining was quantified using the Attune NxT Flow Cytometer with representative images showing the staining intensity in the KO population (pink histogram, dashed line) compared to the WT cells (green histogram, solid line). Histograms with dotted lines represent secondary antibody-only controls in both WT and KO cells. Antibody dilutions used: ab133642** at 1/1050, ab166610** at 1/237, ab167414** at 1/277, ab207303** at 1/999, ABCD_AU751**at 1/5, ABCD_AU752** at 1/850, ABCD_AW383** at 1/35, ARP59239_P050 at 1/5000, ARP96977_P050 at 1/5000, AVARP00015_T100 at 1/1000, MAB1268* at 1/500, NBP1-47957* at 1/100, GTX112707 at 1/810, 66135-1-Ig* at 1/1600, 82918-1-RR** at 1/8000, A0409 at 1/1000, 702047** at 1/500, 711079** at 1/500. *Monoclonal antibody; **Recombinant antibody.

For flow cytometry, Hep G2 WT and *SERPINA1* KO cells were labelled with distinct fluorescent dyes and combined at a 1:1 ratio. Both cell lines were fixed, permeabilised and blocked in the same tube prior to antibody staining to reduce bias. Eighteen A1AT antibodies were then evaluated, with fluorescent intensity assessed using the Attune NxT flow cytometer. Antibody staining in both WT and KO line was then quantified using FlowJo software, with representative histograms presented in Figure 4.

In conclusion, we have screened eighteen A1AT commercial antibodies by western blot, immunoprecipitation and flow cytometry by comparing the signal produced using human Hep G2 WT and *SERPINA1* KO cells. High-quality and renewable antibodies capable of successfully detecting A1AT were identified.

### Limitations

Inherent limitations are associated with the antibody characterization platform used in this study. Firstly, the YCharOS project focuses on renewable (recombinant and monoclonal) antibodies and does not test all commercially available A1AT antibodies. YCharOS partners provide approximately 80% of all renewable antibodies, but some top-cited polyclonal antibodies may not be available through these partners. We encourage readers to consult vendor documentation to identify the specific antigen each antibody is raised against, where such information is available.

Secondly, the YCharOS effort employs a non-biased approach that is agnostic to the protein for which antibodies have been characterized. The aim is to provide objective data on antibody performance without preconceived notions about how antibodies should perform or the molecular weight that should be observed in western blot. As the authors are not experts in A1AT, only a brief overview of the protein’s function and its relevance in disease is provided. A1AT experts are invited to analyse and interpret observed banding patterns in western blots. Thirdly, YCharOS experiments are not performed in replicates primarily due to the use of multiple antibodies targeting various epitopes. Once a specific antibody is identified, it validates the protein expression of the intended target in the selected cell line, confirms the lack of protein expression in the KO cell line and supports conclusions regarding the specificity of the other antibodies. All experiments are performed using master mixes, and meticulous attention is paid to sample preparation and experimental execution. In instances where antibodies yield no signal, a repeat experiment is conducted following titration. Additionally, our independent data is performed subsequently to the antibody manufacturers internal validation process, therefore making our characterization process a repeat.

Lastly, as comprehensive and standardized procedures are respected, any conclusions remain confined to the experimental conditions and cell line used for this study. The use of a single cell type for evaluating antibody performance poses as a limitation, as factors such as target protein abundance significantly impact results. Additionally, the use of cancer cell lines containing gene mutations poses a potential challenge, as these mutations may be within the epitope coding sequence or other regions of the gene responsible for the intended target. Such alterations can impact the binding affinity of antibodies. This represents an inherent limitation of any approach that employs cancer cell lines.

## Methods

The standardized protocols used to carry out this KO cell line-based antibody characterisation platform was established and approved by a collaborative group of academics, industry researchers and antibody manufacturers. The detailed materials and step-by-step protocols used to characterize antibodies in western blot, immunoprecipitation and immunofluorescence are openly available on Protocols.io (DOI: dx.doi.org/10.21203/rs.3.pex-2607/v1).

### Antibodies

All A1AT antibodies are listed in Table 2, together with their corresponding Research Resource Identifiers (RRID), to ensure antibodies are cited properly. ^17^ Secondary antibodies used in this study are provided in Table 3. To ensure consistency with manufacturer recommendations and account for proprietary formulations (where antibody concentrations are not disclosed), antibody usage is reported as dilution ratios rather than absolute concentrations.

**Table 2.** Summary of the Alpha-1-antitrypsin antibodies tested.

Company	Catalogue number	Lot number	RRID (Antibody registry)	Clonality	Clone ID	Host	Stock concentration (μg/mL)	Vendor recommended applications
Abcam	ab133642 **	1014123-6	AB_3668811	Recombinant Monoclonal	EPSISR16	Rabbit	105	IP, WB
Abcam	ab166610 **	1082313-4	AB_3665876	Recombinant Monoclonal	EPR9090	Rabbit	237	ICC/IF, WB
Abcam	ab167414 **	1091979-5	AB_3665875	Recombinant Monoclonal	EPR10832(B)	Rabbit	277	IP, WB
Abcam	ab207303 **	1088914-2	AB_3665874	Recombinant Monoclonal	EPR17087-50	Rabbit	999	ICC/IF, IHC, IP, WB
ABCD Antibodies	ABCD_AU751 **	10/06/2024	AB_3712954	Recombinant Monoclonal	SCFV4B12	Rabbit	5	-
ABCD Antibodies	ABCD_AU752 **	10/06/2024	AB_3712955	Recombinant Monoclonal	SCFV9D5	Rabbit	85	-
ABCD Antibodies	ABCD_AW383 **	10/06/2024	AB_3712956	Recombinant Monoclonal	anti-A1AT-2H2	Rabbit	35	-
Aviva Systems Biology	ARP59239_P050	QC30148-43483	AB_3668851	Polyclonal	-	Rabbit	500	WB
Aviva Systems Biology	ARP96977_P050	QC67938-43131	AB_3668852	Polyclonal	-	Rabbit	500	WB
Aviva Systems Biology	AVARP00015_T100	QC0199-20150424	AB_841679	Polyclonal	-	Rabbit	1000	IHC, WB
Bio-Techne	MAB1268 *	202808	AB_2301508	Monoclonal	202808	Mouse	500	ICC/IF, IHC, IP, WB
Bio-Techne	NBP1-47957 *	F002	AB_10010955	Monoclonal	OTI9A1	Mouse	1000	FC, IHC, IP, WB
GeneTex	GTX112707	40492	AB_11161999	Polyclonal	-	Rabbit	810	IHC, WB
Proteintech	66135-1-Ig *	10003372	AB_2881534	Monoclonal	1A9G6	Mouse	1600	ICC/IF, IHC, IP, FC-Intra, WB
Proteintech	82918-1-RR **	23006677	AB_3668844	Recombinant Monoclonal	230109A8	Rabbit	800	IHC, FC-Intra, WB
Sigma-Aldrich	A0409	-	AB_257883	Polyclonal	-	Rabbit	NA	ELISA, WB
Thermo Fisher Scientific	702047 **	2092596	AB_2689472	Recombinant Monoclonal	8H10L18	Rabbit	500	WB
Thermo Fisher Scientific	711079 **	2799570	AB_2662337	Recombinant Polyclonal	-	Rabbit	500	ICC/IF, WB

ELISA = Enzyme-linked immunosorbent assay, FC = Flow cytometry, ICC/IF = Immunocytochemistry/immunofluorescence, IHC = immunohistochemistry, IP = immunoprecipitation, WB = Western blot, NA = Not available.

* Monoclonal antibody.

** Recombinant antibody.

**Table 3.** Summary of the secondary antibodies used.

Company	Secondary antibody	Catalogue number	RRID (Antibody registry)	Clonality	Application	Stock concentration (μg/mL)	Working concentration (μg/mL)
Proteintech	HRP-Goat Anti-Rabbit Secondary Antibody (H+L)	RGAR001	AB_3073505	Recombinant Polyclonal	Western blot	1000	0.1
Proteintech	HRP-Goat Anti-Mouse Secondary Antibody (H+L)	RGAM001	AB_3068333	Recombinant Polyclonal	Western blot	1000	0.1
Abcam	Veriblot	ab131366	AB_2892718	Not specified	Immunoprecipitation	40	0.04
Proteintech	CoraLite-Plus-647-Goat Anti-Rabbit Secondary Antibody (H+L)	RGAR005	AB_3073509	Recombinant Polyclonal	Flow cytometry	500	0.625
Proteintech	CoraLite-Plus-647-Goat Anti-Mouse Secondary Antibody (H+L)	RGAM005	AB_3073503	Recombinant Polyclonal	Flow cytometry	500	0.625

### Cell culture

All cell lines used in this study are listed in Table 1, alongside their corresponding RRIDs, to ensure proper citation. ^18^ Cells were cultured in Minimum Essential Medium (MEM) Eagle (Sigma-Aldrich #M0325-500ML) supplemented with 10% fetal bovine serum (Thermo Fisher Scientific #A5256801), 1% sodium pyruvate (Thermo Fisher Scientific #11360070), and 1% antibiotic/antimycotic solution (Capricorn Scientific #AAS-B). All cell lines used in this study were routinely tested for mycoplasma contamination and were confirmed to be mycoplasma-free.

### CRISPR/Cas9 genome editing

The Hep G2 *SERPINA1* KO clone was generated using low-passage cells. Prior to ribonucleoprotein transfection, 20,000 cells were plated in each well of a 48-well plate and incubated overnight to allow attachment. in vitro guide RNA was generated with the HighYield T7 sgRNA synthesis kit (Jena Bioscience #RNT-105), followed by incubation with DNase I-XT (New England Biolabs #M0570) and quick CIP (New England Biolabs #M0525) to remove DNA and 5′ phosphorylation respectively. RNA was purified using the Monarch spin RNA cleanup kit (New England Biolabs #T2040) and 125ng of guide RNA (target sequence: CACTCACGATGAAATCCTGG) was combined with 625ng of spCas9. Ribonucleoprotein transfection was then carried out using the Lipofectamine CRISPRMAX Cas9 transfection reagent (Thermo Fisher Scientific #CMAX000008) according to the manufacturer’s protocol. The culture medium was replaced the following day, and single-cell isolation was performed on day three. Single cells were plated into 96-well plates and clonally expanded.

### Antibody screening by western blot

Hep G2 WT and *SERPINA1* KO cells were washed three times in Hanks’ balanced salt solution (HBSS) (Capricorn Scientific #HBSS-2A) and serum deprived for 48-hours in MEM media without phenol red (Thermo Fisher Scientific #51200046) supplemented with 1% sodium pyruvate, 1% antibiotic/antimycotic solution and 1% L-glutamine (Thermo Fisher Scientific #A2916801). Culture medium was then collected and centrifuged for 500 × *g*, for 10 minutes at 4°C to eliminate cells and larger contaminants, then for 4500 × *g*, for 10 minutes at 4°C to eliminate smaller contaminants. Conditioned medium was then concentrated by centrifugation at 4000 × *g* for 30 minutes at 4°C using Amicon Ultra 15mL centrifugal filters with a 3 kDa molecular weight cut off (Sigma Aldrich #UFC900396). Culture media was then supplemented with 1× protease inhibitor cocktail (Cell Signaling Technology #7012).

For lysate preparation, Hep G2 WT and *SERPINA1* KO cells were washed three times in phosphate buffered saline (PBS) (Thermo Fisher Scientific #70011044) and lysed in RIPA buffer containing 1× of protease inhibitor cocktail, sodium orthovanadate and phenylmethylsulfonyl fluoride (Santa Cruz Biotechnology #sc-24948). Lysates were sonicated (40% amplitude for 5 seconds) three times and incubated for 30 minutes on ice prior to centrifugation at 20,000 × *g* for 1 hour at 4°C.

Protein concentration was quantified using the Pierce BCA protein assay (Thermo Fisher Scientific #23225) and 30 μg of protein was used for both protein lysates and concentrated cell culture medium. Samples were combined with Laemmli sample buffer (Bio-Rad #1610747) containing 2-mercaptoethanol (final concentration 355 mM) (Sigma Aldrich #M7522) before being heated at 65°C for 10 minutes. Samples were then loaded in precast 4-20% WedgeWell Tris-Glycine Plus midi gels (Thermo Fisher Scientific #WTG42020BOX) alongside Prime-Step prestained broad range protein ladder (BioLegend #773302). SDS-PAGE was then performed in SureLock Tandem Midi Gel tanks (Thermo Fisher Scientific #STM1001) and run at 200V for 1 hour with Tris/Glycine/SDS buffer (Bio-Rad #1610772). Proteins were then transferred to 0.2μm supported nitrocellulose membranes (Cytiva #10600015) using a Criterion blotter with plate electrodes (Bio-Rad #17004070) run at 85V for 45 minutes. Proteins on the blot were then visualised with Ponceau S staining (Thermo Fisher Scientific #161470250) which was scanned to show alongside individual western blots. Blots were blocked with 5% milk for 1 hour in Tris-buffered saline containing 1% Tween 20 (TBST) (Thermo Fisher Scientific #J77500.K2). Primary antibodies were then incubated overnight at 4°C in 5% milk TBST with gentle shaking. Following three ten-minute washes with TBST, horseradish peroxidase (HRP) conjugated secondary antibodies were incubated at a dilution of 1/10000 (0.1μg/mL) in TBST with 5% milk for 1 hour at room temperature followed by three ten-minute washes with TBST. Membranes were then incubated with either Pierce ECL (Thermo Fisher Scientific #32106) for 1 minute or Clarity Western ECL substrate (Bio-Rad #1705061) for 5 minutes prior to detection with the ImageQuant LAS 4000.

### Antibody screening by immunoprecipitation

Antibody-bead conjugates were prepared by adding 2 μg of antibody to 1 mL of Pierce IP Lysis Buffer (25 mM Tris-HCl pH 7.4, 150 mM NaCl, 1 mM EDTA, 1% NP-40 and 5% glycerol) (Thermo Fisher Scientific #87788) in a microcentrifuge tube, together with 30 μL of protein A (for rabbit antibodies) or protein G (for mouse antibodies) (Thermo Fisher Scientific #10002D and #10004D respectively). Tubes were rocked for 1 hour at 4°C followed by two washes to remove unbound antibody. Culture media from Hep G2 WT were collected as described in the western section above. 0.5 mL aliquots at 1mg/mL of culture medium were incubated with an antibody-bead conjugate for 18 hours at 4°C. The unbound fractions were collected, and beads were subsequently washed three times with 1.0 mL of IP buffer and processed for SDS-PAGE and western blot on precast midi 4-20% Tris-Glycine polyacrylamide gels.

### Antibody screening by flow cytometry

Hep G2 WT and *SERPINA1* KO cells were detached, and five million cells were labelled with CellTracker green or violet fluorescent dyes, respectively (Thermo Fisher Scientific, #C7025 and #C10094). WT and KO cells were then centrifuged at 300 × *g*, for 10 minutes and resuspended in PBS containing 1% bovine serum albumin (BSA) (Sigma-Aldrich, A9647). Cell populations were then combined at a 1:1 ratio, centrifuged and fixed on ice for 20 minutes using 800 μL of 4% PFA in PBS (Thermo Fisher Scientific #J19943.K2). Following fixation, 1.2 mL of 1% BSA in PBS was added to the tube, vortexed and centrifuged at 600 × *g* for 15 minutes at 4°C. Cells were then permeabilised in 400 μL PBS with 0.1% Saponin (Sigma-Aldrich #558255) for 10 minutes at room temperature, centrifuged at 600 × *g* for 15 minutes at 4°C and then blocked with 5% goat serum (Sigma-Aldrich #G6767), 1% BSA, 0.1% saponin in PBS for 30 minutes on ice. After the blocking step 400,000 cells were aliquoted into individually labelled tubes, centrifuged at 600 × *g* for 15 minutes at 4°C and incubated in 150 μL of 1% BSA, 0.1% Saponin PBS with primary A1AT antibodies for 30 minutes on ice. 500 μL of 1% BSA, 0.1% saponin PBS was then added to each tube, vortexed and centrifuged at 600 × *g* for 15 minutes at 4°C. Cells were then incubated with their corresponding Multi-rAb CoraLite ^®^ Plus 647 secondary antibodies (0.83 μg/ml) (Proteintech #RGAR005 and #RGAM005) in 150 μL of 1% BSA, 0.1% saponin PBS for 30 minutes on ice. 500 μL of 1% BSA, 0.1% saponin PBS was then added to each tube, vortexed and centrifuged 600 × *g* for 15 minutes at 4°C.

Tubes were then resuspended in 1 ml of 1% BSA in PBS and data was acquired using the Attune NxT flow cytometer. Data was analysed using FlowJo with the following gates. The cell population was first gated on FSC-A vs SSC-A, within that gate single cells were selected by FSC-A vs FSC-H and then KO and WT cells were isolated by BL1-A vs VL1-A using a quadrat gate. Quantification of antibody staining was then observed in the RL1-A channel and histograms merged to demonstrate the staining intensity between the two populations compared to the two secondary only controls. The figure was then assembled using Adobe Illustrator 2024.

## Data Availability

Zenodo: Dataset for the Alpha-1-antitrypsin antibody screening study, this contains the underlying data included in a study which characterized eighteen commercially available antibodies against Alpha-1-antitrypsin (SERPINA1) by immunoblot (Western blot), immunoprecipitation and flow cytometry, using a knockout based validation. This corresponds to all the data within the figures for this article. https://doi.org/10.5281/zenodo.17925190. Data are available under the terms of the Creative Commons Attribution 4.0 International license (CC-BY 4.0).
