# Supplementary material for: A guide to selecting high-performing antibodies for Alpha-1-antitrypsin (UniProt ID: P01009) for use in western blot, immunoprecipitation and flow cytometry
Source: F1000Res. 2026 Jan 19;15:73. [Version 1] doi: 10.12688/f1000research.175969.1 (PMC12963825; doi:10.12688/f1000research.175969.1)
Supplement: Peer review history

# Reviewer report: Reviewer response for version 1

**DOI:** 10.5256/f1000research.193994.r459390

**Published:** 2026-03-05

**Authors:** Sian E. Evans

**Affiliations:** 1 University of Leicester, Leicester, England, UK

The data note describes the characterisation and specification of 18 commercially available antibodies for Alpha-1-antitrypsin (A1AT) for use in western blot, immunoprecipitation and flow cytometry.

 The aim is to assess antibody specificity for A1AT using the Hep G2 cell line, and to compare readouts from wild-type and CRISPR/Cas9 knockout (KO) cells, using standardised experimental protocols that are openly available online.  

 The results and discussion section is concise and coherent. All images and tables are presented clearly. The authors justify their choice of using the Hep G2 cell line and reference the database they used to identify it. 

 A couple of minor suggestions would be:

 1. To include a table or image which demonstrates the distinction between successful and unsuccessful antibody performance in each of the techniques described. I have come across this in other data notes of this kind and found it extremely useful when interpreting the data.

 2. To demonstrate how you confirmed the successful KO of SERPINA1. 

 3. Further explain why two different ECL reagents were used.

Are sufficient details of methods and materials provided to allow replication by others?

Yes

Is the rationale for creating the dataset(s) clearly described?

Yes

Are the datasets clearly presented in a useable and accessible format?

Yes

Are the protocols appropriate and is the work technically sound?

Yes

Reviewer Expertise:

Using multiplexed immunofluorescence techniques on patient-derived explants, cell biology, and assay development.

I confirm that I have read this submission and believe that I have an appropriate level of expertise to confirm that it is of an acceptable scientific standard.

# Reviewer report: Reviewer response for version 1

**DOI:** 10.5256/f1000research.193994.r451626

**Published:** 2026-02-06

**Authors:** Steve Jean

**Affiliations:** 1 Université de Sherbrooke,, Sherbrooke, QC, Canada

In this manuscript by Cooper et al., the authors use standardized approaches to assess the specificity of 18 commercial Alpha‑1‑antitrypsin antibodies across three applications (western blot, immunoprecipitation, and FACS). The manuscript is clear, well structured, and contributes to the broader antibody characterization effort led by the YCharOS initiative. I am particularly impressed that all original data, along with detailed protocols, are openly available on a Zenodo webpage. I have only two minor suggestions that could further improve the clarity and interpretation of the presented data:

1. Provide the genomic sequence of the KO clone used in the study. CRISPR/Cas9 editing commonly results in diverse indels or insertions. Including the sequence of the affected genomic region in the *SERPINA1* KO clone used throughout the manuscript would help readers fully interpret the antibody‑validation results and understand the nature of the knockout.
2. Clarify exposure conditions for western blot images. From the raw data, it appears that multiple exposures were acquired on the LAS4000 and that most antibodies were tested simultaneously. Indicating whether all figures were generated using the same exposure time, or specifying the exposure times when they differ, would facilitate more accurate comparison across antibodies.

Are sufficient details of methods and materials provided to allow replication by others?

Yes

Is the rationale for creating the dataset(s) clearly described?

Yes

Are the datasets clearly presented in a useable and accessible format?

Yes

Are the protocols appropriate and is the work technically sound?

Yes

Reviewer Expertise:

Cell biology, proteomics, membrane trafficking

I confirm that I have read this submission and believe that I have an appropriate level of expertise to confirm that it is of an acceptable scientific standard.
